# 554 Implementation of the Abbreviated Burn Specific Pain and Anxiety Scale

**DOI:** 10.1093/jbcr/irae036.188

**Published:** 2024-04-17

**Authors:** Jonny Harms, Morgan Treftz, Paige Dispalatro, Courtney Bode, Hailey Worthington

**Affiliations:** University of Kansas Medical Center, Kansas City, KS; University of Kansas Medical Center, Kansas City, KS; University of Kansas Medical Center, Kansas City, KS; University of Kansas Medical Center, Kansas City, KS; University of Kansas Medical Center, Kansas City, KS

## Abstract

**Introduction:**

This project targeted the use of the Abbreviated Burn Specific Pain and Anxiety Scale (BSPAS) in patients 18 or greater with 10-50% TBSA burns undergoing dressing change and/or therapy. The BSPAS is recognized by the ABA and was implemented to improve both patient care and nursing practice. Currently, in our standard of care there are no tools available to specifically assess anxiety related to pain. This can drastically impact the overall care of burn patients in various stages of care including treatment, recovery, and rehabilitation.

**Methods:**

Pre-data surveys were used to assess nursing staff’s perceptions of incorporating the BSPAS into practice. Per the survey, nurses felt they did not have a consistent scale to measure the burn patient’s pain and anxiety. Our implementation of the BSPAS consisted of 5 questions asked by the nurse to better understand the patient’s pain and anxiety prior to treatment.

**Results:**

A post-implementation survey revealed that nurses felt the BSPAS was a consistent and effective way to better understand and communicate a patient’s pain and anxiety related to the anticipation of burn care.

**Conclusions:**

Our sample size was small due to limited burn patient admissions during the pilot phase of this project. The current nurse residency group has continued to implement and evaluate the use of the BSPAS to improve the quality and overall standard of care within our unit.

**Applicability of Research to Practice:**

Our project can help burn units better understand patient’s anxiety and pain related to burn dressing changes and therapy.

